# Optimal Management of High-Risk T1G3 Bladder Cancer: A Decision Analysis

**DOI:** 10.1371/journal.pmed.0040284

**Published:** 2007-09-25

**Authors:** Girish S Kulkarni, Antonio Finelli, Neil E Fleshner, Michael A. S Jewett, Steven R Lopushinsky, Shabbir M. H Alibhai

**Affiliations:** 1 Division of Urology, Department of Surgical Oncology, University of Toronto, Toronto, Ontario, Canada; 2 Department of Health Policy, Management and Evaluation, University of Toronto, Toronto, Ontario, Canada; 3 Division of General Internal Medicine and Clinical Epidemiology, University of Toronto, Toronto, Ontario, Canada; Mount Sinai Medical Center, United States of America

## Abstract

**Background:**

Controversy exists about the most appropriate treatment for high-risk superficial (stage T1; grade G3) bladder cancer. Immediate cystectomy offers the best chance for survival but may be associated with an impaired quality of life compared with conservative therapy. We estimated life expectancy (LE) and quality-adjusted life expectancy (QALE) for both of these treatments for men and women of different ages and comorbidity levels.

**Methods and Findings:**

We evaluated two treatment strategies for high-risk, T1G3 bladder cancer using a decision-analytic Markov model: (1) Immediate cystectomy with neobladder creation versus (2) conservative management with intravesical bacillus Calmette-Guérin (BCG) and delayed cystectomy in individuals with resistant or progressive disease. Probabilities and utilities were derived from published literature where available, and otherwise from expert opinion. Extensive sensitivity analyses were conducted to identify variables most likely to influence the decision. Structural sensitivity analyses modifying the base case definition and the triggers for cystectomy in the conservative therapy arm were also explored. Probabilistic sensitivity analysis was used to assess the joint uncertainty of all variables simultaneously and the uncertainty in the base case results. External validation of model outputs was performed by comparing model-predicted survival rates with independent published literature. The mean LE of a 60-y-old male was 14.3 y for immediate cystectomy and 13.6 y with conservative management. With the addition of utilities, the immediate cystectomy strategy yielded a mean QALE of 12.32 y and remained preferred over conservative therapy by 0.35 y. Worsening patient comorbidity diminished the benefit of early cystectomy but altered the LE-based preferred treatment only for patients over age 70 y and the QALE-based preferred treatment for patients over age 65 y. Sensitivity analyses revealed that patients over the age of 70 y or those strongly averse to loss of sexual function, gastrointestinal dysfunction, or life without a bladder have a higher QALE with conservative therapy. The results of structural or probabilistic sensitivity analyses did not change the preferred treatment option. Model-predicted overall and disease-specific survival rates were similar to those reported in published studies, suggesting external validity.

**Conclusions:**

Our model is, to our knowledge, the first of its kind in bladder cancer, and demonstrated that younger patients with high-risk T1G3 bladder had a higher LE and QALE with immediate cystectomy. The decision to pursue immediate cystectomy versus conservative therapy should be based on discussions that consider patient age, comorbid status, and an individual's preference for particular postcystectomy health states. Patients over the age of 70 y or those who place high value on sexual function, gastrointestinal function, or bladder preservation may benefit from a more conservative initial therapeutic approach.

## Abstract 2

Using a Markov model, Shabbir Alibhai and colleagues develop a decision analysis comparing cystectomy with conservative treatment for high-risk superficial bladder cancer depending on patient age, comorbid conditions, and preferences.

## Editors' Summary

**Background.:**

Every year, about 67,000 people in the US develop bladder cancer. Like all cancers, bladder cancer arises when a single cell begins to grow faster than normal, loses its characteristic shape, and moves into surrounding tissues. Most bladder cancers develop from cells that line the bladder (“transitional” cells) and most are detected before they spread out of this lining. These superficial or T1 stage cancers can be removed by transurethral resection of bladder tumor (TURBT). The urologist (a specialist who treats urinary tract problems) passes a small telescope into the bladder through the urethra (the tube through which urine leaves the body) and removes the tumor. If the tumor cells look normal under a microscope (so-called normal histology), the cancer is unlikely to return; if they have lost their normal appearance, the tumor is given a “G3” histological grade, which indicates a high risk of recurrence.

**Why Was This Study Done?:**

The best treatment for T1G3 bladder cancer remains controversial. Some urologists recommend immediate radical cystectomy— surgical removal of the bladder, the urethra, and other nearby organs. This treatment often provides a complete cure but can cause serious short-term health problems and affects long-term quality of life. Patients often develop sexual dysfunction or intestinal (gut) problems and sometimes find it hard to live with a reconstructed bladder. The other recommended treatment is immunotherapy with bacillus Calmette-Guérin (BCG, bacteria that are also used to vaccinate against tuberculosis). Long-term survival is not always as good with this conservative treatment but it is less likely than surgery to cause short-term illness or to reduce quality of life. In this study, the researchers have used decision analysis (a systematic evaluation of the important factors affecting a decision) to determine whether immediate cystectomy or conservative therapy is the optimal treatment for patients with T1G3 bladder cancer. Decision analysis allowed the researchers to account for quality-of-life factors while comparing the health benefits of each treatment for T1G3 bladder cancer.

**What Did the Researchers Do and Find?:**

Using a decision analysis model called a Markov model, the researchers calculated the months of life gained, and the quality of life expected to result, from each of the two treatments. To estimate the life expectancy (LE) associated with each treatment, the researchers incorporated the published probabilities of various outcomes of each treatment into their model. To estimate quality-adjusted life expectancy (QALE, the number of years of good quality life), they incorporated “utilities,” measures of relative satisfaction with outcomes. (A utility of 1 represents perfect health; death is assigned a value of 0, and outcomes considered less than ideal, but better than death, fall in between). For a sexually potent 60-year-old man with bladder cancer but no other illnesses, the average LE predicted by the model was nearly eight months longer with immediate cystectomy than with conservative treatment (both LEs predicted by this model matched those seen in clinical trials); the average QALE with cystectomy was 4.2 months longer than with conservative treatment. Having additional diseases decreased the benefit of immediate cystectomy but the treatment still gave a longer LE until the patient reached 70 years old, when conservative treatment became better. For QALE, this change in optimal treatment appeared at age 65. Finally, conservative treatment gave a higher QALE than immediate cystectomy for patients concerned about preserving sexual function or averse to living with intestinal problems or a reconstructed bladder.

**What Do These Findings Mean?:**

As with all mathematical models, these results depend on the assumptions included in the model. In particular, because published probability and utility values are not available for some of the possible outcomes of the two treatments, the LE and QALE calculations could be inaccurate. Also, assigning numerical ratings to life experiences is generally something of a simplification, which could affect the reliability of the QALE (but not the LE) results. Nevertheless, these findings provide useful guidance for urologists trying to balance the benefits of immediate cystectomy or conservative treatment against the potential short-term and long-term effects of these treatments on patients' quality of life. Specifically, the results indicate that decisions on treatment for T1G3 bladder cancer should be based on a consideration of the patient's age and any coexisting disease coupled with detailed discussions with the patient about their attitudes regarding the possible health-related effects of cystectomy.

**Additional Information.:**

Please access these Web sites via the online version of this summary at http://dx.doi.org/10.1371/journal.pmed.0040284.

- MedlinePlus encyclopedia page on bladder cancer (in English and Spanish)
- Information for patients and professionals from the US National Cancer Institute on bladder cancer (in English and Spanish)
- Information for patients on bladder cancer from the UK charity Cancerbackup
- Online course on Decision Analysis in Health Care from George Mason University

## Introduction

In North America, more than 65,000 patients are diagnosed with bladder cancer annually [1]. The majority of cases are superficial transitional cell carcinoma (TCC), which are usually managed by transurethral resection (TURBT). High-grade superficial TCC that invades the lamina propria of the bladder is staged as T1G3 and may further be divided into high-risk (multifocal and/or associated carcinoma in situ [CIS]) or low-risk (solitary without associated CIS) categories [2].

Currently, two main treatment options exist for the treatment of high-risk T1G3 bladder cancer. Immediate radical cystectomy provides the best opportunity for cure. Cystectomy, however, is associated with short-term morbidity, including perioperative death, and may impact negatively on quality of life (QOL), because patients often develop long-term sexual dysfunction and/or complications relating to urinary diversion. The second up-front option consists of repeated instillations of intravesical immunotherapy in the form of bacillus Calmette-Guérin (BCG). Responders to BCG retain their bladder and are thereby spared cystectomy and its associated complications, thus maintaining their QOL. However, BCG therapy may be associated with poorer survival outcomes compared to cystectomy for a number of reasons. First, a number of T1G3 tumors are understaged at TURBT and are in fact muscle invasive (stage T2). Muscle invasive lesions carry a higher risk of metastases and death than T1 lesions and are thus best treated with cystectomy. Second, tumor recurrence and/or tumor progression to muscle invasion can occur while on BCG therapy. In fact, the 3-y risk of recurrence and progression to muscle invasion following BCG is 80% and 35%–48%, respectively [3]. The high progression rates of high-risk T1G3 tumors and their potential for progression have consequently led some urologists to favor immediate cystectomy.

Controversy exists as to the optimal treatment of patients with high-risk T1G3 TCC. Any gain in life expectancy (LE) from better disease control with cystectomy must be weighed against a potential loss in QOL. Additionally, any survival benefits associated with cystectomy may be counterbalanced by competing risks of mortality in older patients or those with significant comorbid medical illnesses. One technique for comparing treatment strategies that enables incorporation of both LE and QOL and considers variables such as age or comorbidity is decision analysis. To date, no decision analyses have been performed for T1G3 bladder cancer. We sought to identify the ideal strategy for patients with high-risk T1G3 bladder cancer using a Markov decision-analytic model.

## Methods

### Overview

We constructed a Markov model (TreeAge Pro Suite 2005) to determine whether a patient with high-risk T1G3 bladder cancer should undergo one of two strategies: (1) immediate nerve-sparing cystectomy with orthotopic neobladder creation; (2) conservative therapy with potential for delayed cystectomy. Radiotherapy was not considered part of the usual treatment algorithm for these tumors. We used 6 mo cycle lengths in order to model BCG therapy. Outcomes assessed were LE and quality-adjusted life expectancy (QALE).

### Base Case Definition

Our base case was a 60-y-old, otherwise well, compliant and sexually potent man with newly diagnosed high-risk T1G3 TCC. The T1G3 diagnosis was assumed to be bladder-confined and based on a TURBT containing muscularis propria, indicating an adequate resection [4].

### Model Design

**Figure 1:**
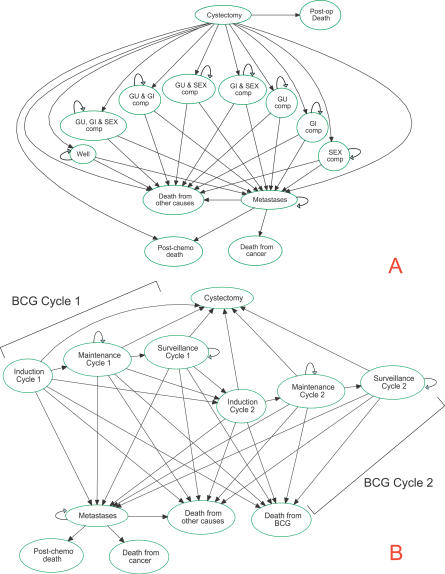
State Transition Diagrams for Immediate Cystectomy (A) and Conservative Therapy with BCG (B) Abbreviations: GI comp, gastrointestinal complications (diarrhea, abdominal pain, or metabolic complications secondary to ileal resection); GU comp, genitourinary complications (incontinence, sepsis, ureteral stenosis, or recurrent urinary tract infections); Mets, metastases; SEX comp, sexual complications (impotence).

Figure 1 depicts the Markov state transition diagram for both strategies. Patients undergoing immediate cystectomy (Figure 1A) may die postoperatively secondary to complications from cystectomy or may die of other causes in each cycle. Survivors entered one of eight mutually exclusive health states that are combinations of the three major potential long-term complications of cystectomy: sexual dysfunction, genitourinary dysfunction, and gastrointestinal dysfunction. The probability of short-term complications (“surgically related morbidity”) and long-term sequelae after cystectomy were modeled independent of pathological stage [5]. Disease recurrence after cystectomy was defined as metastases. The probability of developing metastatic disease was based on the patient's cystectomy pathological stage. We assumed that all individuals with lymph node positive disease or metastases were eligible for adjuvant [6] or palliative [7–9] platinum-based chemotherapy, respectively. We also assumed that the risk of metastases was dependent on tumor stage and nodal status but independent of the local treatment strategy.

Patients undergoing conservative therapy received an induction course of six instillations of BCG followed by 2.5 y of maintenance BCG (Figure 1B) [10]. Patients recurring with T1G3 disease or CIS were considered BCG failures and were eligible for a second cycle of BCG therapy. Individuals with two BCG failures or those who progressed to muscle invasion underwent cystectomy. At any point during BCG therapy or its associated surveillance phase, patients were at risk of occult metastatic disease, recurrent disease, or death from BCG complications or other causes. As with cystectomy, we assumed that the risk of metastases was dependent on tumor stage and nodal status. This assumption enabled us to incorporate both understaged and progressive T1G3 lesions into the model. Progressive tumors had a higher T stage and nodal metastases risk at cystectomy compared to true T1G3 lesions. Patients requiring delayed cystectomy entered postoperative health states identical to those undergoing immediate cystectomy. Movements between health states for both treatment strategies were dictated by published probabilities.

### Data Sources

We performed a MEDLINE search (1966–2005) to identify relevant papers from which probabilities and utilities could be obtained. For probabilities, the following text words or medical subject headings (MeSH) were utilized: “bladder neoplasms”, “T1G3”, “cystectomy”, “BCG”, “Bacille Calmette-Guerin” and “follow-up studies”. For utilities, we cross-referenced bladder neoplasms with the text words “utility” or “utilities” and the MeSH headings “decision” and “quality-adjusted life years.” A manual search of the reference lists from identified studies, meta-analyses, and review articles was performed to ensure that important articles were not missed. In cases where estimates of probabilities or utilities could not be obtained, expert opinion was sought from uro-oncologists.

### Probabilities and Utilities

#### Probabilities.

Actuarial life-tables were used to estimate the age-specific risk of dying from other causes independent of health state or treatment strategy [11]. Probabilities for both treatment strategies were obtained from both randomized controlled trials and retrospective cohort series and are listed in Table S1. Specifically, each study was analyzed for every possible probability in the model, and values were extracted and tabulated accordingly. Consequently, multiple sources for each probability estimate were often obtained. Rates taken from time-to-event analyses were converted to semi-annual probabilities assuming an exponential distribution [*P*(x) = 1 − *e* ^−*uT*^]. Final probabilities were calculated by weighting each article's probabilities by its sample size. However, if one or more articles for a given probability was of higher quality (e.g., a randomized controlled trial) than other sources, its published value was chosen. Plausible ranges for each probability were defined by the extremes of published values for the given probability in situations where multiple studies were pooled. In this manner, plausible ranges for each probability were conservative and included the values from all papers which reported that probability. In situations where only one paper served as the source of a probability, the plausible range was defined by the published 95% confidence interval, where available, or ± 50% of the chosen probability value otherwise.

#### Utilities and treatment complications.

Published utilities for bladder cancer patients were not available. Consequently, we derived the utility of an uncomplicated, post-cystectomy health state from a standard gamble involving 25 urologists and urology trainees at our institution. Comparable health state preferences from populations with similar health issues were used for the remaining utilities in the model. These were obtained from the Tufts-New England Medical Center Cost-Effectiveness Analysis registry (http://www.tufts-nemc.org/cearegistry/data/default.asp). We felt extrapolation of utilities was reasonable given that health state preferences are defined primarily by patient symptoms, which are often similar regardless of disease site. Nevertheless, given the uncertainty in our utility estimates, we also reported LE outcomes in addition to QALE outcomes in order to assess our model's results in the absence of utilities.

The utility of impotence and genitourinary complications were obtained from a recent comprehensive decision analysis for prostate cancer [12]. The utility of long-term gastrointestinal complications post-cystectomy was taken from patients who have undergone ileal reservoir creation since these individuals, like ileal neobladder patients, experience bowel resection, reanastomosis, and reconstruction and thus possibly similar long-term sequelae. The short-term, postoperative utility of undergoing cystectomy was adapted from utilities measured for abdominal hysterectomy, colostomy creation for nonsevere trauma and radical prostatectomy [13–15]. The utility associated with cystectomy was not modified based on the timing of cystectomy (immediate versus delayed while on BCG therapy), since current evidence does not suggest a change in patient preference for cystectomy based on its timing.

The impact of metastases was reflected in terms of downstream treatment (i.e., chemotherapy), increased risk of disease-specific mortality, and utilities associated with metastatic disease and its treatment. The utility of metastases, regardless of the initial treatment strategy, was assigned based on a patient's responsiveness to chemotherapy (i.e., ability to achieve a complete or partial remission). The responsive metastases health state was assigned a utility weight of 0.62. The unresponsive metastases health state was assigned a utility weight of 0.30. In other words, time in either of these health states was weighted, in a multiplicative manner, by the health state's respective utility. Both of these values were extrapolated from the breast cancer literature [16,17]. We chose to extrapolate from breast cancer because the metastatic landing zones (i.e., bones and viscera) are common between breast and bladder cancer and thus, both patient groups would be expected to experience similar symptomatology.

We derived utilities for induction BCG, maintenance BCG, and surveillance cystoscopy based on published utilities associated with outpatient, moderately invasive procedures such as cardiac catheterization [18]. We did not model the morbidity of living with undiagnosed locally recurrent bladder cancer because such lesions are usually asymptomatic. The utility of diagnosis of a recurrent lesion on BCG therapy was incorporated in the utility for a cystoscopy (0.997) and the utility of treatment of recurrent lesions was incorporated by assigning a disutility for TURBT of −0.06. Recurrences that required subsequent BCG therapy were subject to the utilities of the BCG health states as listed in Table S1.

We applied transitional penalties (disutilities) to account for the short term complications and inconvenience of procedures in both arms (i.e., cystectomy, chemotherapy, BCG, and TURBT). Similar to utilities, disutilities subtract from a given health state's baseline utility value. Disutilities for cystectomy complications were based on conditions commonly observed postoperatively [19–22]. The disutility of chemotherapy (i.e., the inconvenience of, and symptomatology arising from, chemotherapy), adapted from cancer patients undergoing chemotherapy for breast and small cell lung cancer, was −0.36 [16,23]. We modeled additional morbidity in the form of chemotherapy-specific complications such as death, febrile neutropenia, severe mucositis, etc. using a disutility value for nonlethal chemotherapy complications of −0.54 [17]. Death in all circumstances was assigned a utility of 0. Uncertainty around utility estimates was set to include estimates from all studies reporting a similar utility or, where multiple studies were unavailable, ± 50% of the base case estimate.

### Model Assumptions

First, we assumed that long-term complications following radical cystectomy are static (time-independent) and not correlated with one another. Second, we assumed that upper tract and urethral recurrences are likely to develop independent of the treatment choices offered and thus will not bias results in either direction. Third, we assumed that only one recurrence on conservative therapy could occur per 6 mo cycle. This assumption allowed for two recurrences in the first year on BCG or, alternatively, two potential BCG failures in the first year of conservative therapy. Fourth, we assumed equal progression and recurrence rates while in the maintenance and surveillance health states of each respective BCG cycle and that risks of recurrence and progression were higher in patients requiring a second cycle of BCG [24]. Fifth, we assumed that all utility values were time- and age-independent.

### Comorbidity Adjustment

Rate ratios for comorbidity from two retrospective bladder cancer studies, one assessing long-term, overall mortality [25] for patients with invasive bladder cancer and the other postoperative (short-term) mortality [26] for cystectomy patients, modified the probability of mortality from non-bladder cancer causes and the probability of postcystectomy mortality in a multiplicative manner, respectively.

### Validation

We assessed the ability of our model to reproduce medium-term (5-y) and long-term (10- or 15-y) overall survival and disease-specific survival rates in both treatment arms by comparing model-generated survival estimates with those published in large studies that were separate from those used to generate model probabilities.

### Sensitivity Analyses

#### One-way sensitivity analyses.

To assess the stability of our results, one-way sensitivity analyses were performed by varying each probability and utility between 0 and 1 (the broadest range possible). Thresholds were identified for sensitive variables either within or outside their plausible ranges. Also, by reporting both LE and QALE outcomes, we were able to assess the model's robustness without the uncertainty of our utility estimates.

#### Structural sensitivity analyses.

Although we assumed that patients treated with initial BCG would move on to cystectomy after suffering two BCG failures over a minimum of one year [2,27], cystectomy for patients suffering BCG failures in the first six months of therapy (early failures) or cystectomy following a single BCG failure are recognized alternative strategies [2,27]. We explicitly explored these alternative scenarios in our model using structural sensitivity analyses. We also tested our modeling assumption limiting the number of recurrences to two in one year by allowing a third recurrence in the first year of therapy and determining the impact on model outcomes.

Finally, we assessed the impact of varying our base case definition. Specifically, we determined outcomes for women as the base case (instead of men) and for men with preexisting erectile dysfunction (instead of potent men). These modifications increased the generalizability of our model by including important patient populations who were not fully represented by our original base case definition.

#### Probabilistic sensitivity analyses.

We performed probabilistic sensitivity analysis using 1,000 second-order Monte Carlo simulations. This process allowed us to address the joint uncertainty of all model parameters simultaneously and provided a more accurate estimate of the average health outcomes of each strategy. To perform this analysis, we modeled all of our input variables as distributions. We assumed that “transition probabilities” (probabilities of moving from one health state to another) followed an exponential distribution and calculated corresponding transition rates and standard errors for each study. These values were used to estimate the parameters of a gamma distribution using the method of moments [28]. We selected gamma distributions because, like transition rates, the range of possible values is bounded by zero and infinity. Thus, in each run of the model, we randomly sampled each transition rate from that parameter's gamma distribution and converted this rate back to a transition probability. We modeled “event probabilities” (probabilities of a one-time event) and utility scores by using beta distributions (bounded by 0 and 1) defined by the estimate and standard error of each parameter. In each run of the model, we sampled each event probability and utility from that respective parameter's beta distribution.

## Results

### Base Case LE and QALEs

The average (mean) LE of a 60-y-old, potent man with high-risk disease and no comorbidities undergoing immediate cystectomy was 14.29 y. With conservative management, the average LE was 13.63 y. Thus, immediate cystectomy was preferred by 0.66 y. With the addition of utilities, the immediate cystectomy strategy yielded an average QALE of 12.32 y and remained preferred over conservative therapy by 0.35 QALY (quality-adjusted life years).

### Base Case Validation

For radical cystectomy outcomes, our model predicted 5- and 10-y overall survival rates of 80% and 62%, respectively, which are consistent with the respective rates of 78% and 58% reported by Stein et al. [29]. The 5-y, model-predicted, overall survival rate in the conservative therapy arm was 81%, which mirrors published literature [3,10,30]. The 15-y predicted overall survival rate in the conservative therapy arm was 41%, similar to the published long-term result of 37% for high-risk T1 and/or CIS patients [3].

For immediate cystectomy, the 5- and 10-y disease-specific survival rates generated by the model were 91% and 82%, respectively, which are similar to the respective 90% [31] and 83% [32] rates reported in the literature. Our model produced 5- and 10-y disease-specific survival rates of 90% and 79%, respectively, for conservative therapy compared to observed rates of 87% and 75%, respectively [30,33].

### One-Way Sensitivity Analyses

For the base case (60-y-old males), the preferred therapy was sensitive to four probabilities and three utilities (Tables 1 and 2, respectively). Patient age at diagnosis and comorbidity were the most influential probabilities (see below). Other important variables included the conditional probability of developing a progressive tumor given a recurrence and the utilities for impotence, gastrointestinal complications, and urinary diversion (i.e., the postcystectomy state).

**Table 1:**
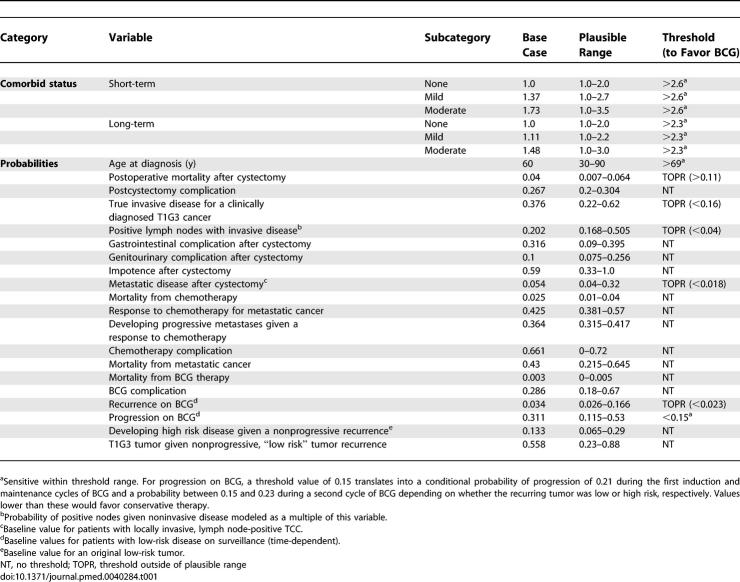
One-Way Sensitivity Analysis Results for a 60-Year-Old Potent Male: Comorbid Status and Probabilities

**Table 2:**
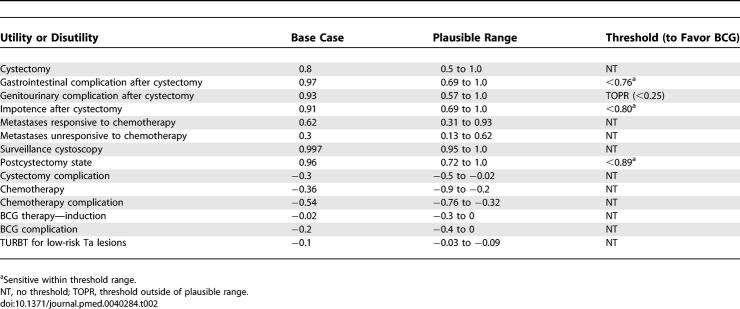
One-Way Sensitivity Analysis Results for a 60-Year-Old Potent Male: Utilities and Disutilities

### Impact of Age and Comorbidity

**Figure 2:**
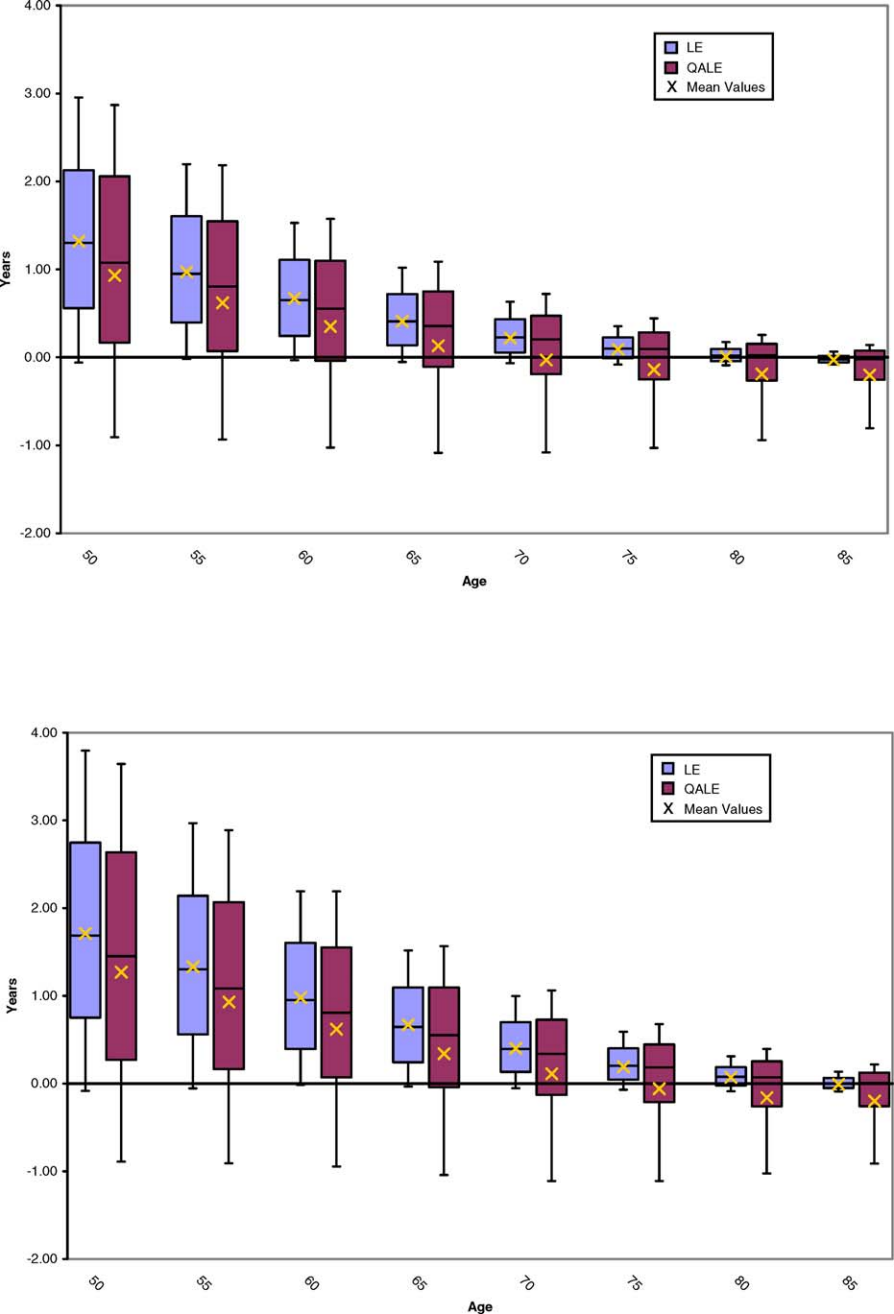
Box Plots of Incremental Gain in LE and QALE with Immediate Cystectomy by Age for Male (Upper Plot) and Female Patients (Lower Plot) The region in the shaded boxes of both plots represents the interquartile range. The solid line within boxes is the median incremental gain, whereas the mean value is presented as an “×”. Upper and lower whiskers indicate the 10th and 90th percentiles, respectively.

Health outcomes for men at various age intervals are displayed in Figure 2. Preferred treatment strategies were most susceptible to change between the ages of 75 and 85 y for mean LE outcomes, and 65 to 75 y for mean QALE outcomes and most consistent outside of these age ranges. After the age of 80 y, the treatment with the greatest mean LE changed from immediate cystectomy to conservative management. Likewise, as men approach the age of 70 y, the treatment with the greatest mean QALE changed to conservative therapy.

**Figure 3:**
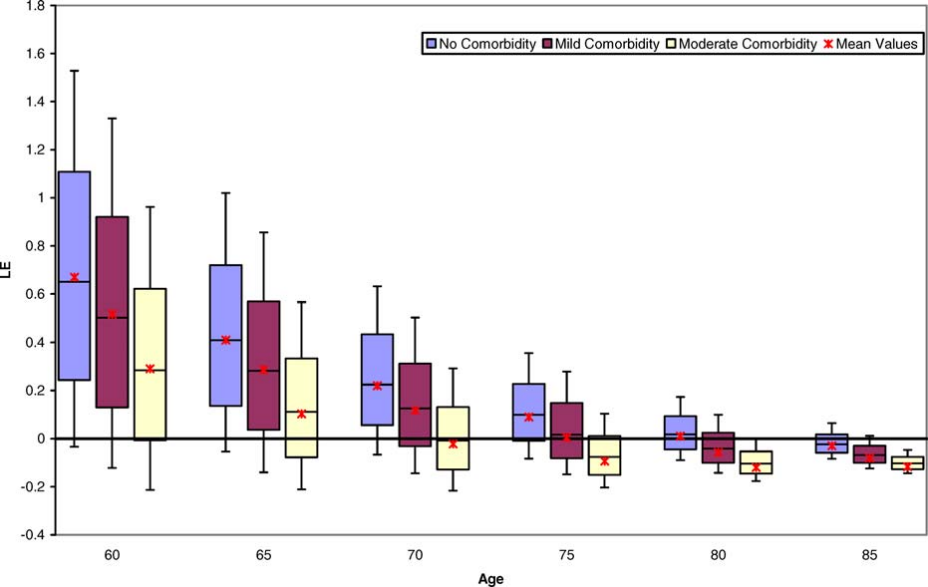
Box Plot of Gain in LE with Immediate Cystectomy by Age and Comorbid Status for Male Patients The region in the shaded boxes represents the interquartile range. The solid line within boxes is the median incremental gain, whereas the mean value is presented as an asterisk. Upper and lower whiskers indicate the 10th and 90th percentiles, respectively.

**Figure 4:**
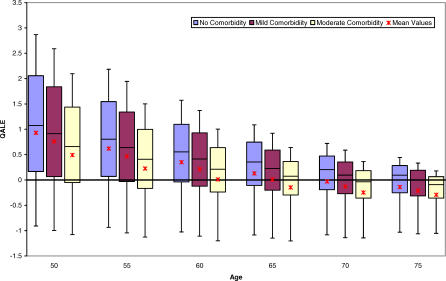
Box plot of Gain in QALE with Immediate Cystectomy by Age and Comorbid Status for Male Patients The region in the shaded boxes represents the interquartile range. The solid line within boxes is the median incremental gain, whereas the mean value is presented as an asterisk. Upper and lower whiskers indicate the 10th and 90th percentiles, respectively.

Incorporating comorbidity into the LE model did not significantly alter the mean results for the base case (male, age 60) or for very young or very old patients (Figure 3). Where mean LE outcomes were considered, comorbid status was most important for patients between the ages of 70 and 80 y. Specifically, immediate cystectomy yielded a higher mean LE only for 70-y-old men with no or mild comorbidity and 75-y-old men without comorbid illness. For individuals aged 80 y, a treatment decision could not be made based on LE for those with no comorbidity given the near equivalent mean LE of both treatment strategies. With respect to mean QALE outcomes (Figure 4), comorbidity most influenced outcomes for men between the ages of 60 and 70 y. For the base case, cystectomy was favored in situations where comorbidity was absent or mild. However, for a 60-y-old man with moderate comorbidity, immediate cystectomy was associated with such a small incremental mean QALE gain (0.04 QALY) that a preferred treatment could not be identified with certainty. Similar uncertainty existed for a 70-y-old man with no comorbidity. Individuals above age 60 with moderate comorbidity had a higher average QALE with conservative therapy. Assessing the impact of comorbidity in the LE model revealed similar trends (Figure 4).

### Structural Sensitivity Analyses

The impact of various triggers for cystectomy during BCG therapy was assessed via structural sensitivity analyses (Table 3). A total of three scenarios were compared: (1) The base case in which cystectomy was triggered after two BCG failures, defined as a T1G3 recurrence after a second cycle of BCG; (2) cystectomy after a single BCG failure, defined as a T1G3 recurrence after a single cycle of BCG; or (3) cystectomy after an early BCG failure (during the first 6 mo of BCG therapy). In all three scenarios, the mean LE and QALE estimates for conservative therapy changed minimally and did not alter the preferred treatment.

**Table 3:**
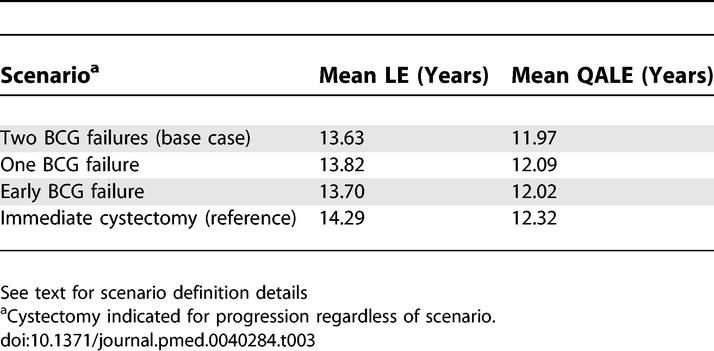
Impact of Alternative Triggers for Radical Cystectomy during Initial BCG Therapy

Varying the base case definition also did not alter the model's health outcomes. For example, female patients had similar, albeit slightly larger, average LE and QALE outcomes compared to males (Figure 2, lower box plot). Likewise, the mean QALE for patients with preexisting erectile dysfunction was 0.62 y higher with immediate cystectomy compared to initial conservative management (12.99 y versus 12.37 y). Allowing a third recurrence on BCG in the first year of therapy also did not materially influence the results, with the net mean incremental QALE gain for immediate cystectomy decreasing by only 0.06 y.

### Probabilistic Sensitivity Analyses

Jointly varying the input parameters in a probabilistic sensitivity analysis did not alter the treatment decision. The mean LE for the base case via probabilistic sensitivity analysis was 14.61 y (interquartile range [IQR] 13.81–15.63 y) for immediate cystectomy and 13.89 y (IQR 13.03–14.92 y) for up-front BCG. Likewise, the base case mean QALEs were 12.62 y (IQR 11.35–14.49 y) and 12.24 y (IQR 11.03–13.69 y) for radical and conservative management, respectively. With respect to QALE, 74% of simulations favored an up-front cystectomy, which can be considered moderate to good effectiveness. In other words, we are 74% confident that cystectomy is the preferred choice for high-risk T1G3 lesions in base case patients. Assuming a minimal clinically important difference of 0.2 QALE (2.4 mo) for one strategy to be truly favored, 67% of simulations still favored immediate cystectomy, 21% favored BCG, and 12% of simulations were indifferent (that is, neither strategy was better by at least 0.2 QALE). For 50-y-old patients, 78% of simulations favored cystectomy, whereas 55% of simulations favored BCG for 85-y-old patients. Similar results were found for LE simulations (89% for immediate cystectomy for 50-y-old patients; 66% for conservative therapy for 85-y-old patients).

**Figure 5:**
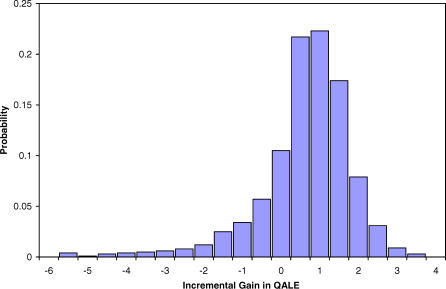
Incremental QALE Gains for the Base Case The histogram depicts the distribution of results of 1,000 Monte Carlo simulations for 60-y-old male patients without comorbidity. Positive values indicate a larger QALE for immediate cystectomy as compared to conservative therapy. The distribution is skewed to the left.

Probabilistic sensitivity analysis also enabled calculation of credible intervals for our comparisons of the two treatment strategies. These are represented in Figures 2, 3, and 4 in the form of box-and-whisker plots. The uncertainty implied by the credible intervals, coupled with the fact that immediate cystectomy is favored in the majority (74%) of simulations for the base case, indicate a skewed distribution of incremental gains as depicted in Figure 5. Incremental gains with cystectomy are skewed because simulations with a high postoperative death rate postcystectomy result in large gains for conservative management for a minority of patients.

## Discussion

We found that for an otherwise healthy, 60-y-old sexually potent man with high-risk T1G3 bladder cancer, the mean LE was 7.9 mo higher if he decided to undergo immediate radical cystectomy instead of conservative management. Consideration of QOL led to a slightly smaller gain of 4.2 mo in favor of early cystectomy but did not alter the preferred treatment option. Comorbidity was most influential on the treatment decision for patients between the ages of 60 and 70 y. One-way sensitivity analyses revealed that patients over the age of 70 or those strongly averse to loss of sexual function, gastrointestinal dysfunction, or life without a bladder had a higher QALE with conservative therapy. Neither structural sensitivity analyses nor probabilistic sensitivity analyses altered the optimal base case treatment strategy.

This work represents, to our knowledge, the first systematic evaluation of the two main treatment strategies for high-risk T1G3 TCC of the bladder. While the best means of comparing two or more treatment strategies is a randomized controlled trial, such a methodology may not be feasible for T1G3 cancer, given the difficulties inherent in randomizing patients to invasive versus less invasive therapies in surgical trials [34]. Indeed, we are not aware of any planned or ongoing trial examining this issue. These pragmatic issues therefore position a decision analytic model as an important methodology to address the T1G3 TCC controversy.

We have identified several important factors that are relevant to decision-making in the setting of high-risk T1G3 bladder cancer. Patient age and comorbid status were the most important variables driving the decision in our model. Our analysis also identified three key utilities that may potentially sway the treatment decision for patients. Specifically, patients who place great value on preserving sexual function and/or those who are strongly averse to gastrointestinal symptomatology or to living with a urinary diversion may fare better with conservative management. While our model assumed a cystectomy technique incorporating nerve sparing and creation of an orthotopic ileal neobladder, deviation from this approach (i.e., urostomy and/or non-nerve-sparing) could diminish the utility associated with urinary diversion and/or alter the probability of postoperative sexual complications, and thereby alter the treatment decision. Reports published to date, however, do not support significant QOL differences based on the type of urinary diversion [35,36]. Nevertheless, these studies do not measure utilities and it remains possible that small QOL differences may generate differences in utility values that could then impact the results of our model. However, the extent to which alternative surgical techniques affect bladder cancer patient utilities has not been studied and is a focus for future research. In addition to the factors identified in this study, patient anxiety, fear, and circumstances unique to each case (e.g., social or financial concerns), which are not addressed by our model, should also be considered in the T1G3 decision-making process.

We assumed that patients embarking on a bladder conservation treatment strategy could undergo salvage cystectomy after developing a T1G3 recurrence after two cycles of BCG. Since the timing of cystectomy while on BCG therapy is contentious, we performed structural sensitivity analyses by modifying the triggers for cystectomy to reflect the varied practice patterns of urologists (Table 3). Regardless of the point at which cystectomy was offered to a patient on BCG therapy, immediate cystectomy conferred a higher incremental LE and QALE gain compared to conservative management for our base case. Alternative base case scenarios (e.g., preexisting sexual dysfunction, female patient, or multiple recurrences) also did not alter the preferred treatment, further pointing to the robustness of our results.

Our base case was diagnosed with a high-risk T1G3 lesion after a single TURBT. Current evidence suggests that the standard of care for T1G3 disease is shifting away from a single TURBT to re-resection with a second TURBT prior to embarking on a treatment decision [37,38]. A repeat TURBT could help identify patients with invasive disease and thereby decrease the proportion of patients who inappropriately receive BCG. Improved patient selection with two TURBT procedures would be reflected in diminished recurrence and progression rates and thus improved outcomes while on conservative therapy. We were able to examine the benefit of a second TURBT after a T1G3 diagnosis via a sensitivity analysis on the probability of occult invasive disease (Table 1). According to our model, this proportion would have to be reduced to 16%, a potentially reasonable target after two TURBTs, to alter the treatment decision. Further long-term outcome data after two TURBTs are necessary to ultimately validate this cut-point. Improvements in conservative therapy could also alter the treatment decision by decreasing recurrence and progression rates. Our model supported this possibility by identifying threshold recurrence and progression values below which conservative therapy was favored.

The insights gained from our model are consistent with international guidelines on the treatment of T1G3 TCC of the bladder [2,27]. They also provide additional clarification that has not previously been reported. In particular, our study is the first, to our knowledge, to quantify the age ranges that may potentially be associated with suboptimal health outcomes following immediate cystectomy for high-risk T1G3 bladder cancer. The available guidelines also list comorbid status as an important determinant guiding treatment decisions, but provide no details to clarify decision-making. Our analysis refines this concept by demonstrating that comorbidity is most likely to affect the preferred treatment strategy for patients who are between ages 70 and 80 and between 60 and 70 for LE and QALE outcomes, respectively.

Validity assessment suggests that our model is accurate since the survival rates it generated concurred with those in the published literature. Nevertheless, important limitations to this study exist. First, many of the studies from which probabilities were drawn were retrospective in nature with possible selection biases. For example, long-term cystectomy series may have excluded patients with significant comorbidity, whereas concurrent BCG series may have included such individuals. Along with disease-specific external validation, which may theoretically address some of the selection biases inherent in using overall survival as an outcome, we also attempted to deal with this limitation by identifying the highest-quality studies and by conducting extensive sensitivity analyses. Second, a lack of utility data for bladder cancer patients forced us to either extrapolate utilities from similar disease states or use clinician-derived utilities. Although we feel these utilities are representative, and were varied widely in sensitivity analyses to reflect the uncertainty inherent to extrapolation, they may not fully characterize the preferences of bladder cancer patients. For this reason, we also reported life expectancy outputs from our model, which were independent of utilities and gave similar results. Nevertheless, elicitation of utilities from bladder cancer patients, particularly those utilities to which the model was sensitive, is an important area of future research. Third, we defined our base case after a single TURBT. As we alluded to above, many urologists are now offering a second TURBT soon after the initial resection to better risk-stratify patients. Repeat TURBT, however, is a relatively new approach in the treatment of high-risk T1G3 tumors, and thus data on outcomes after a second resection are only now emerging [38–40]. Consequently, a lack of available long-term outcomes data precluded us from using a T1G3 diagnosis based on a second TURBT in our base case. As this information becomes increasingly available, we will be able to incorporate it into future iterations of our model. As a novel treatment approach, there is also little evidence that suggests that repeat TURBT has been routinely adopted by most urologists, which suggests that our model in its current form is relevant to contemporary clinical practice. Nevertheless, we were able to model the effect of a second TURBT by varying our probability of occult invasive disease in one-way sensitivity analyses and have reported these analyses. Fourth, our model only allowed for a maximum of two disease recurrences in one year. Although rare, three recurrences may occur in a year on conservative therapy. Since the preferred treatment was cystectomy rather than conservative management, this limitation is unlikely to impact materially on our results. This notion was supported via structural sensitivity analyses in which we explored three recurrences on BCG in the first year of therapy. Fifth, we did not explicitly consider noncompliance or dose-reduced BCG protocols in our model, since most studies reporting recurrence and progression rates with BCG therapy incorporate patients with poor compliance or dose reductions [10,41]. Nevertheless, although diminished compliance with BCG has not been shown to affect recurrence or progression rates [42], some clinicians may recommend immediate cystectomy if patient compliance with BCG is a concern.

Despite these limitations, the paucity of sensitive variables illustrates the robustness of our results. Furthermore, a mean difference of 7.9 mo of LE and 4.2 mo of QALE for the base case analysis is considered moderate to substantial by medical decision makers. While these values may seem small, LE gains in decision models are diluted across patients and represent average values, thus making a gain of this magnitude considerable. The actual clinical significance of a gain in life expectancy from a decision model can be determined by comparing the obtained gain with results derived from widely accepted interventions in similar populations [43]. The LE results generated from our model compare favorably to other interventions in adults with established disease. For example, routine beta-blocker therapy in men at moderate risk of recurrence after a myocardial infarction is an established treatment modality and is associated with a gain in LE of approximately 4.1 mo [44]. Likewise, myocardial revascularization with coronary artery bypass grafting for coronary artery disease confers a 1–7, 0–8, and 4–14 mo gain in LE for single, double, and triple vessel disease, respectively [18]. The gains in LE from both of these generally accepted and widely practiced interventions are in line with those generated by our model, supporting the importance of our model's outcomes.

In conclusion, our model demonstrates that an otherwise well 60-y-old patient with high-risk T1G3 TCC of the bladder would have a higher LE and QALE with immediate cystectomy. The decision to pursue immediate cystectomy versus conservative therapy should be based on discussions that consider patient age, comorbid status, and an individual's preference for particular postcystectomy health states. Patients who place great emphasis on preserving sexual function or who are strongly averse to living with chronic gastrointestinal symptoms or a neobladder may maximize their health outcomes with a bladder-sparing regimen.

## Supporting Information

Table S1 Model Semiannual Probabilities and Utilities Semiannual probabilities and the utilities/disutilities used to populate the decision model.
(128 KB DOC)

## Abbreviations

BCG: bacille Calmette-Guérin
CIS: carcinoma in situ
IQR: interquartile range
LE: life expectancy
QALE: quality-adjusted life expectancy
QALY: quality-adjusted life years
QOL: quality of life
TCC: transitional cell carcinoma
TURBT: transurethral resection of bladder tumor
