# Supplementary material for: Optimal Management of High-Risk T1G3 Bladder Cancer: A Decision Analysis
Source: PLoS Med. 2007 Sep 25;4(9):e284. doi: 10.1371/journal.pmed.0040284 (PMC1989749; doi:10.1371/journal.pmed.0040284)
Supplement: Table S1 — Semiannual probabilities and the utilities/disutilities used to populate the decision model. (128 KB DOC) [file pmed.0040284.st001.doc]

SUPPLEMENT

The following table depicts the semi-annual probabilities and the utilities/disutilities used to populate the decision model.

Supplement, Table 1: Model Semi-annual Probabilities and Utilities.

| **DESCRIPTION** | **Subcategory** | **Prob** | **Plausible Range** | **References** |
| --- | --- | --- | --- | --- |
| **CYSTECTOMY ARM** |  |  |  |  |
| Postoperative mortality |  | 0.04 | 0.007-0.064 | [1, 2] |
| Probability of a peri-operative cystectomy complication |  | 0.267 | 0.2-0.304 | [3, 4] |
| True invasive disease for a clinically diagnosed T1G3 cancer |  | 0.376 | 0.22-0.62 | [5-8] |
| Positive lymph nodes given invasive disease |  | 0.202 | 0.168-0.505 | [5, 9, 10] |
| Positive lymph nodes given noninvasive disease |  | 0.06 | 0.05-0.15 | [5, 9, 10] |
| GI complication |  | 0.316 | 0.09-0.395 | [11] |
| GU complication |  | 0.1 | 0.075-0.256 | [3, 11-13] |
| Impotence |  | 0.59 | 0.4-1.0 | [12, 14-16] |
| Metastatic disease after cystectomy | Locally non-invasive, lymph node negative | 0.010 | 0.008-0.059 | [5, 6, 9] |
|  | Locally invasive, lymph node negative | 0.016 | 0.012-0.095 | [9] |
|  | Locally non-invasive, lymph node positive | 0.037 | 0.028-0.219 | [9] |
|  | Locally invasive, lymph node positive | 0.054 | 0.04-0.32 | [5, 9] |
| Mortality from metastatic cancer |  | 0.43 | 0.215-0.645 | [17] |
| **BCG ARM** |  |  |  |  |
| Mortality from BCG therapy |  | 0.003 | 0-0.005 | [18] |
| BCG complication |  | 0.286 | 0.18-0.67 | [19, 20] |
| Recurrence on BCG - *Cycle 1* | Induction – High risk | 0.263 | 0.201-1.0 | [21-24] |
|  | Induction – Low risk | 0.089 | 0.068-0.435 | [23, 25-27] |
|  | Maintenance/Surveillance – High risk | 0.091 | 0.070-0.444 | [21, 22, 24] |
|  | Maintenance/Surveillance – Low risk | 0.034 | 0.026-0.166 | [19, 25, 27] |
| Recurrence on BCG - *Cycle 2* | Induction – High risk | 0.367 | 0.281-1.0 | [24, 28] |
|  | Induction – Low risk | 0.27 | 0.206-1.0 | [27] |
|  | Maintenance/Surveillance – High risk | 0.155 | 0.119-0.757 | [24, 28] |
|  | Maintenance/Surveillance – Low risk‡ | 0.27 | 0.206-1.0 | - |
| Progression on BCG given a recurrence - *Cycle 1* | Induction – High risk | 0.47 | 0.174-0.801 | [22] |
|  | Induction – Low risk | 0.311 | 0.115-0.53 | [19, 20, 29] |
|  | Maintenance/Surveillance – High risk | 0.47 | 0.174-0.801 | [22] |
|  | Maintenance/Surveillance – Low risk | 0.311 | 0.115-0.53 | [19, 20, 29] |
| Progression on BCG given a recurrence - *Cycle 2* | Induction – High risk | 0.357 | 0.132-0.608 | [28] |
|  | Induction – Low risk‡ | 0.311 | 0.115-0.53 | - |
|  | Maintenance/Surveillance – High risk | 0.5 | 0.185-0.852 | [28] |
|  | Maintenance/Surveillance – Low risk‡ | 0.311 | 0.115-0.53 | - |
| Developing high risk disease given a non-progressive recurrence | If original tumor low risk | 0.133 | 0.065-0.29 | [24, 29] |
|  | If original tumor high risk | 0.548 | 0.268-1.0 | [24, 28] |
| T1G3 tumor given non-progressive, “low risk” tumor recurrence |  | 0.548 | 0.23-0.88 | [20, 27, 29] |
| **COMORBIDITY*** |  |  |  |  |
| Short-term# | None | 1.0 | 1.0-3.5 | [1] |
|  | Mild | 1.37 | 1.0-3.5 | [1] |
|  | Moderate | 1.73 | 1.0-3.5 | [1] |
| Long-term | None | 1.0 | 1.0-3.0 | [30] |
|  | Mild | 1.11 | 1.0-3.0 | [30] |
|  | Moderate | 1.48 | 1.0-3.0 | [30] |
| **CHEMOTHERAPY SPECIFIC** |  |  |  |  |
| Mortality from chemotherapy |  | 0.025 | 0.01-0.04 | [17, 31, 32] |
| Response to chemotherapy for metastatic cancer† |  | 0.425 | 0.381-0.57 | [17, 31, 33] |
| Developing progressive metastases given a response to chemotherapy |  | 0.364 | 0.315-0.417 | [17, 31, 34] |
| Chemotherapy complication |  | 0.661 | 0-0.72 | [17, 31] |
| **(DIS)UTILITIES** |  |  |  |  |
| Cystectomy |  | 0.8 | 0.5-1.0 | [35-37] |
| GI complication after cystectomy |  | 0.97 | 0.69-1.0 | [38] |
| GU complication after cystectomy |  | 0.93 | 0.57-1.0 | [39] |
| Impotence after cystectomy |  | 0.91 | 0.69-1.0 | [39] |
| Metastases responsive to chemotherapy |  | 0.62 | 0.31-0.93 | [40] |
| Metastases unresponsive to chemotherapy |  | 0.3 | 0.13-0.62 | [41] |
| Surveillance cystoscopy |  | 0.997 | 0.95-1.0 | - |
| Post-cystectomy state |  | 0.96 | 0.72-1.0 | - |
| (Cystectomy complication) |  | -0.3 | -0.5 to -0.02 | [42-45] |
| (Chemotherapy) |  | -0.36 | -0.9 to -0.2 | [40, 46] |
| (Chemotherapy complication) |  | -0.54 | -0.76 to -0.32 | [41] |
| (BCG therapy – induction)‡ |  | -0.02 | -0.3-0 | - |
| (BCG complication)‡ |  | -0.2 | -0.4-0 | - |
| (TURBT for low risk Ta lesions) |  | -0.06 | -0.03 to -0.09 | [37] |

*Relative risk ratios

#Patients with a Charlson Comorbidity score of at least 2 were assumed to represent moderate comorbidity. Mild comorbidity was Interpolated

†Complete or partial remission

‡Expert Opinion

# Prob = probability

REFERENCES

1. Elting LS, Pettaway C, Bekele BN, Grossman HB, Cooksley C, et al. (2005) Correlation between annual volume of cystectomy, professional staffing, and outcomes: A statewide, population-based study. Cancer 104(5): 975-984.

2. Birkmeyer JD, Siewers AE, Finlayson EV, Stukel TA, Lucas FL, et al. (2002) Hospital volume and surgical mortality in the united states. N Engl J Med 346(15): 1128-1137.

3. Hautmann RE, de Petriconi R, Gottfried HW, Kleinschmidt K, Mattes R, et al. (1999) The ileal neobladder: Complications and functional results in 363 patients after 11 years of followup. J Urol 161(2): 422-7; discussion 427-8.

4. Cookson MS, Chang SS, Wells N, Parekh DJ, Smith JA,Jr. (2003) Complications of radical cystectomy for nonmuscle invasive disease: Comparison with muscle invasive disease. J Urol 169(1): 101-104.

5. Ghoneim MA, el-Mekresh MM, el-Baz MA, el-Attar IA, Ashamallah A. (1997) Radical cystectomy for carcinoma of the bladder: Critical evaluation of the results in 1,026 cases. J Urol 158(2): 393-399.

6. Bianco FJ,Jr, Justa D, Grignon DJ, Sakr WA, Pontes JE, et al. (2004) Management of clinical T1 bladder transitional cell carcinoma by radical cystectomy. Urol Oncol 22(4): 290-294.

7. Freeman JA, Esrig D, Stein JP, Simoneau AR, Skinner EC, et al. (1995) Radical cystectomy for high risk patients with superficial bladder cancer in the era of orthotopic urinary reconstruction. Cancer 76(5): 833-839.

8. Pagano F, Bassi P, Galetti TP, Meneghini A, Milani C, et al. (1991) Results of contemporary radical cystectomy for invasive bladder cancer: A clinicopathological study with an emphasis on the inadequacy of the tumor, nodes and metastases classification. J Urol 145(1): 45-50.

9. Stein JP, Lieskovsky G, Cote R, Groshen S, Feng AC, et al. (2001) Radical cystectomy in the treatment of invasive bladder cancer: Long-term results in 1,054 patients. J Clin Oncol 19(3): 666-675.

10. Solsona E, Iborra I, Rubio J, Casanova J, Almenar S. (2004) The optimum timing of radical cystectomy for patients with recurrent high-risk superficial bladder tumour. BJU Int 94(9): 1258-1262.

11. Henningsohn L, Steven K, Kallestrup EB, Steineck G. (2002) Distressful symptoms and well-being after radical cystectomy and orthotopic bladder substitution compared with a matched control population. J Urol 168(1): 168-74; discussion 174-5.

12. Kessler TM, Burkhard FC, Perimenis P, Danuser H, Thalmann GN, et al. (2004) Attempted nerve sparing surgery and age have a significant effect on urinary continence and erectile function after radical cystoprostatectomy and ileal orthotopic bladder substitution. J Urol 172(4 Pt 1): 1323-1327.

13. Turner WH, Danuser H, Moehrle K, Studer UE. (1997) The effect of nerve sparing cystectomy technique on postoperative continence after orthotopic bladder substitution. J Urol 158(6): 2118-2122.

14. Zippe CD, Raina R, Massanyi EZ, Agarwal A, Jones JS, et al. (2004) Sexual function after male radical cystectomy in a sexually active population. Urology 64(4): 682-5; discussion 685-6.

15. Schoenberg MP, Walsh PC, Breazeale DR, Marshall FF, Mostwin JL, et al. (1996) Local recurrence and survival following nerve sparing radical cystoprostatectomy for bladder cancer: 10-year followup. J Urol 155(2): 490-494.

16. Brendler CB, Steinberg GD, Marshall FF, Mostwin JL, Walsh PC. (1990) Local recurrence and survival following nerve-sparing radical cystoprostatectomy. J Urol 144(5): 1137-40; discussion 1140-1.

17. von der Maase H, Hansen SW, Roberts JT, Dogliotti L, Oliver T, et al. (2000) Gemcitabine and cisplatin versus methotrexate, vinblastine, doxorubicin, and cisplatin in advanced or metastatic bladder cancer: Results of a large, randomized, multinational, multicenter, phase III study. J Clin Oncol 18(17): 3068-3077.

18. Rawls WH, Lamm DL, Lowe BA, Crawford ED, Sarosdy MF, et al. (1990) Fatal sepsis following intravesical bacillus calmette-guerin administration for bladder cancer. J Urol 144(6): 1328-1330.

19. Martinez-Pineiro JA, Flores N, Isorna S, Solsona E, Sebastian JL, et al. (2002) Long-term follow-up of a randomized prospective trial comparing a standard 81 mg dose of intravesical bacille calmette-guerin with a reduced dose of 27 mg in superficial bladder cancer. BJU Int 89(7): 671-680.

20. Pansadoro V, Emiliozzi P, de Paula F, Scarpone P, Pansadoro A, et al. (2002) Long-term follow-up of G3T1 transitional cell carcinoma of the bladder treated with intravesical bacille calmette-guerin: 18-year experience. Urology 59(2): 227-231.

21. Lamm DL, Blumenstein BA, Crissman JD, Montie JE, Gottesman JE, et al. (2000) Maintenance bacillus calmette-guerin immunotherapy for recurrent TA, T1 and carcinoma in situ transitional cell carcinoma of the bladder: A randomized southwest oncology group study. J Urol 163(4): 1124-1129.

22. Shahin O, Thalmann GN, Rentsch C, Mazzucchelli L, Studer UE. (2003) A retrospective analysis of 153 patients treated with or without intravesical bacillus calmette-guerin for primary stage T1 grade 3 bladder cancer: Recurrence, progression and survival. J Urol 169(1): 96-100; discussion 100.

23. Brausi M, Collette L, Kurth K, van der Meijden AP, Oosterlinck W, et al. (2002) Variability in the recurrence rate at first follow-up cystoscopy after TUR in stage ta T1 transitional cell carcinoma of the bladder: A combined analysis of seven EORTC studies. Eur Urol 41(5): 523-531.

24. Lam JS, Benson MC, O'Donnell MA, Sawczuk A, Gavazzi A, et al. (2003) Bacillus calmete-guerin plus interferon-alpha2B intravesical therapy maintains an extended treatment plan for superficial bladder cancer with minimal toxicity. Urol Oncol 21(5): 354-360.

25. Serretta V, Pavone C, Ingargiola GB, Daricello G, Allegro R, et al. (2004) TUR and adjuvant intravesical chemotherapy in T1G3 bladder tumors: Recurrence, progression and survival in 137 selected patients followed up to 20 years. Eur Urol 45(6): 730-5; discussion 735-6.

26. Holmang S, Johansson SL. (2002) Stage ta-T1 bladder cancer: The relationship between findings at first followup cystoscopy and subsequent recurrence and progression. J Urol 167(4): 1634-1637.

27. Yan Y, Andriole GL, Humphrey PA, Kibel AS. (2002) Patterns of multiple recurrences of superficial (Ta/T1) transitional cell carcinoma of bladder and effects of clinicopathologic and biochemical factors. Cancer 95(6): 1239-1246.

28. O'Donnell MA, Krohn J, DeWolf WC. (2001) Salvage intravesical therapy with interferon-alpha 2b plus low dose bacillus calmette-guerin is effective in patients with superficial bladder cancer in whom bacillus calmette-guerin alone previously failed. J Urol 166(4): 1300-4, discussion 1304-5.

29. Soloway MS, Sofer M, Vaidya A. (2002) Contemporary management of stage T1 transitional cell carcinoma of the bladder. J Urol 167(4): 1573-1583.

30. Chahal R, Sundaram SK, Iddenden R, Forman DF, Weston PM, et al. (2003) A study of the morbidity, mortality and long-term survival following radical cystectomy and radical radiotherapy in the treatment of invasive bladder cancer in yorkshire. Eur Urol 43(3): 246-257.

31. Hainsworth JD, Meluch AA, Litchy S, Schnell FM, Bearden JD, et al. (2005) Paclitaxel, carboplatin, and gemcitabine in the treatment of patients with advanced transitional cell carcinoma of the urothelium. Cancer 103(11): 2298-2303.

32. Loehrer PJ S, Einhorn LH, Elson PJ, Crawford ED, Kuebler P, et al. (1992) A randomized comparison of cisplatin alone or in combination with methotrexate, vinblastine, and doxorubicin in patients with metastatic urothelial carcinoma: A cooperative group study. J Clin Oncol 10(7): 1066-1073.

33. Moore MJ, Winquist EW, Murray N, Tannock IF, Huan S, et al. (1999) Gemcitabine plus cisplatin, an active regimen in advanced urothelial cancer: A phase II trial of the national cancer institute of canada clinical trials group. J Clin Oncol 17(9): 2876-2881.

34. von der Maase H, Sengelov L, Roberts JT, Ricci S, Dogliotti L, et al. (2005) Long-term survival results of a randomized trial comparing gemcitabine plus cisplatin, with methotrexate, vinblastine, doxorubicin, plus cisplatin in patients with bladder cancer. J Clin Oncol 23(21): 4602-4608.

35. Sculpher M. (1998) A cost-utility analysis of abdominal hysterectomy versus transcervical endometrial resection for the surgical treatment of menorrhagia. Int J Technol Assess Health Care 14(2): 302-319.

36. Brasel KJ, Borgstrom DC, Weigelt JA. (1999) Management of penetrating colon trauma: A cost-utility analysis. Surgery 125(5): 471-479.

37. Krahn MD, Mahoney JE, Eckman MH, Trachtenberg J, Pauker SG, et al. (1994) Screening for prostate cancer. A decision analytic view. JAMA 272(10): 773-780.

38. McLeod RS, Churchill DN, Lock AM, Vanderburgh S, Cohen Z. (1991) Quality of life of patients with ulcerative colitis preoperatively and postoperatively. Gastroenterology 101(5): 1307-1313.

39. Alibhai SM, Naglie G, Nam R, Trachtenberg J, Krahn MD. (2003) Do older men benefit from curative therapy of localized prostate cancer? J Clin Oncol 21(17): 3318-3327.

40. Leung PP, Tannock IF, Oza AM, Puodziunas A, Dranitsaris G. (1999) Cost-utility analysis of chemotherapy using paclitaxel, docetaxel, or vinorelbine for patients with anthracycline-resistant breast cancer. J Clin Oncol 17(10): 3082-3090.

41. Brown RE, Hutton J, Burrell A. (2001) Cost effectiveness of treatment options in advanced breast cancer in the UK. Pharmacoeconomics 19(11): 1091-1102.

42. Grann VR, Sundararajan V, Jacobson JS, Whang W, Heitjan DF, et al. (2000) Decision analysis of tamoxifen for the prevention of invasive breast cancer. Cancer J 6(3): 169-178.

43. Gould MK, Dembitzer AD, Doyle RL, Hastie TJ, Garber AM. (1999) Low-molecular-weight heparins compared with unfractionated heparin for treatment of acute deep venous thrombosis. A meta-analysis of randomized, controlled trials. Ann Intern Med 130(10): 800-809.

44. Hamel MB, Phillips RS, Davis RB, Teno J, Connors AF, et al. (2000) Outcomes and cost-effectiveness of ventilator support and aggressive care for patients with acute respiratory failure due to pneumonia or acute respiratory distress syndrome. Am J Med 109(8): 614-620.

45. Nicholson T, McGuire A, Milne R. (2001) Cost-utility of enoxaparin compared with unfractionated heparin in unstable coronary artery disease. BMC Cardiovasc Disord 1: 2.

46. Berthelot JM, Will BP, Evans WK, Coyle D, Earle CC, et al. (2000) Decision framework for chemotherapeutic interventions for metastatic non-small-cell lung cancer. J Natl Cancer Inst 92(16): 1321-1329.
